# Death Zone Weather Extremes Mountaineers Have Experienced in Successful Ascents

**DOI:** 10.3389/fphys.2021.696335

**Published:** 2021-07-05

**Authors:** Robert K. Szymczak, Michał Marosz, Tomasz Grzywacz, Magdalena Sawicka, Marta Naczyk

**Affiliations:** ^1^Department of Emergency Medicine, Faculty of Health Sciences, Medical University of Gdańsk, Gdańsk, Poland; ^2^Institute of Meteorology and Water Management – National Research Institute, Warsaw, Poland; ^3^Institute of Physical Culture, Kazimierz Wielki University in Bydgoszcz, Bydgoszcz, Poland; ^4^Department of Neurology, Faculty of Medicine, Medical University of Gdańsk, Gdańsk, Poland; ^5^Department of Nutritional Biochemistry, Faculty of Health Sciences, Medical University of Gdańsk, Gdańsk, Poland

**Keywords:** altitude, weather, extremes, mountaineering, barometric pressure, temperature, wind

## Abstract

**Background:**

Few data are available on mountaineers’ survival prospects in extreme weather above 8000 m (the Death Zone). We aimed to assess Death Zone weather extremes experienced in climbing-season ascents of Everest and K2, all winter ascents of 8000 m peaks (8K) in the Himalayas and Karakoram, environmental records of human survival, and weather extremes experienced with and without oxygen support.

**Materials and Methods:**

We analyzed 528 ascents of 8K peaks: 423 non-winter ascents without supplemental oxygen (Everest–210, K2–213), 76 ascents in winter without oxygen, and 29 in winter with oxygen. We assessed environmental conditions using the ERA5 dataset (1978–2021): barometric pressure (BP), temperature (Temp), wind speed (Wind), wind chill equivalent temperature (WCT), and facial frostbite time (FFT).

**Results:**

The most extreme conditions that climbers have experienced with and without supplemental oxygen were: BP 320 hPa (winter Everest) vs. 329 hPa (non-winter Everest); Temp –41°C (winter Everest) vs. –45°C (winter Nanga Parbat); Wind 46 m⋅s^–1^ (winter Everest) vs. 48 m⋅s^–1^ (winter Kangchenjunga). The most extreme combined conditions of BP ≤ 333 hPa, Temp ≤ −30°C, Wind ≥ 25 m⋅s^–1^, WCT ≤ −54°C and FFT ≤ 3 min were encountered in 14 ascents of Everest, two without oxygen (late autumn and winter) and 12 oxygen-supported in winter. The average extreme conditions experienced in ascents with and without oxygen were: BP 326 ± 3 hPa (winter Everest) vs. 335 ± 2 hPa (non-winter Everest); Temp −40 ± 0°C (winter K2) vs. −38 ± 5°C (winter low Karakoram 8K peaks); Wind 36 ± 7 m⋅s^–1^ (winter Everest) vs. 41 ± 9 m⋅s^–1^ (winter high Himalayan 8K peaks).

**Conclusions:**

1. The most extreme combined environmental BP, Temp and Wind were experienced in winter and off-season ascents of Everest.
2. Mountaineers using supplemental oxygen endured more extreme conditions than climbers without oxygen.
3. Climbing-season weather extremes in the Death Zone were more severe on Everest than on K2.
4. Extreme wind speed characterized winter ascents of Himalayan peaks, but severely low temperatures marked winter climbs in Karakoram.

## Introduction

The pioneering era to be the first climbers of 8000 meter (8K) peaks ended with the first winter ascent of K2 in January 2021; it was the last 8K peak unclimbed in winter (Benavides, 2021b). Since G. Mallory, E. Norton, and H. Somervell took their first steps above 8000 m as members of the British Everest expedition in 1922 (Somervell, 1938) it has taken climbers almost a century to reach all 14 of the world’s 8K peaks without supplemental oxygen in the climbing and the winter seasons. The first 8K peak climbed without oxygen was Annapurna by the French alpinists Herzog and Lachenal, 1950 (Herzog, 1953). Mount Everest, the highest peak on Earth, was first climbed with oxygen support by Hillary and Norgay, 1953 (Hunt and Hillary, 1953) and without supplemental oxygen by Messner and Habeler, 1978 (Habeler, 1979; Messner, 1979). In 1978 the US alpinist L. Reichardt climbed K2 (8611 m), the last 8K peak unclimbed without oxygen support.

Yet more extreme winter Himalayan mountaineering was launched in February 1980 with the first winter oxygen-supported ascent of Everest by K. Wielicki and L. Cichy (Brniak and Nyka, 1981). Only one climber, Ang Rita Sherpa, has repeated their feat without supplemental oxygen, in December 1987 (West, 1993; Garrido et al., 2019; Salisbury and Hawley, 2020). Manaslu was the first 8K peak climbed in winter without oxygen, by the Polish party of Berbeka and Gajewski, 1984 (Korniszewski, 1984). K2 was the last 8K peak unclimbed in winter, but was finally climbed by a team of 10 Nepalese in January 2021 (Farooq, 2021). One climber, Nirmal Purja, reached K2’s summit without supplemental oxygen (Benavides, 2021b). Between Herzog and Lachenal’s success on Annapurna in 1950 and 2021 more than 6500 times 8K peaks have been climbed without supplemental oxygen: about 4500 ascents in the Himalayas (1950–2020) (Salisbury and Hawley, 2020) and about 2070 in Karakoram (1953–2009) (8000ers, 2021).

Altitude, latitude and season mainly determine climatic conditions such as barometric pressure (BP), air temperature (Temp) and wind speed (Wind) (Whiteman, 2000). The higher the altitude and the latitude, and the colder the season, the lower are BP and Temp (Brunt, 1952; West, 1996; Whiteman, 2000; West et al., 2007c). Summit bids on 8K peaks face most risk when climbers enter the Death Zone, the altitudes above 8000 m where extreme conditions threaten human survival (West et al., 2007a). BP imposes the main limit on physiological performance at high altitude because it determines the critically important partial pressure of inspired oxygen, maximum oxygen uptake and speed of vertical ascent (Pugh et al., 1964; West and Wagner, 1980; West, 1983, 2000; West et al., 1983a, b, 2007b; Sutton et al., 1988; Bailey, 2001; Matthews et al., 2020b). Low temperatures and high winds significantly compound physiological stress at extreme altitudes (Huey et al., 2001; Huey and Eguskitza, 2001; McIntosh et al., 2008; Moore and Semple, 2011, 2012; Moore et al., 2012).

A climber’s maximal and sustainable power in the Death Zone drop to levels where activity becomes impossible and the body’s heat generation plummets, greatly increasing the risk of hypothermia (Havenith, 2010). The combined effect of Temp and Wind on a climber can be expressed by thermal stress indices such as wind chill temperature (WCT) and facial frostbite time (FFT) (Tikuisis and Osczevski, 2003; Osczevski and Bluestein, 2005). Wind chill temperature is calculated as an air temperature that without wind would cause the same steady-state facial heat loss as occurs at a given temperature and wind speed (Osczevski and Bluestein, 2005). Facial frostbite time is defined as the time it takes facial flesh to freeze (Moore and Semple, 2011). Low BP leads to hypoxia and high-altitude illness, low temperatures with high winds determine the risk of hypothermia and frostbite: these are the main environmental factors responsible for most non-traumatic deaths of mountaineers above the base camp at Everest (Firth et al., 2008). On Everest, more than 80% of all climbers’ deaths have occurred in the Death Zone in the summit bid (Firth et al., 2008).

Meteorological data for the conditions endured while climbing in the Death Zone are usually restricted to single weather factors on Everest (West et al., 1983b; West, 1999; Moore and Semple, 2004; Grocott et al., 2010; Moore et al., 2012; Moore and Semple, 2012; Matthews et al., 2020b). The estimated average climbing-season BP on Everest is 333 hPa, dropping to 323 hPa in midwinter (Matthews et al., 2020b). A BP of 329 hPa is the lowest recorded for any ascent of Everest unsupported by oxygen (West, 1993; Matthews et al., 2020b). The most extreme Temp encountered on Everest’s summit was –49°C with Wind as high as 80 m⋅s^–1^ (Matthews et al., 2020a). Everest’s average climbing-season WCT is –45°C, FFT is 7 min; in midwinter WCT is close to –65°C and FFT is less than 1 min (Moore and Semple, 2011). In a previous article (Szymczak et al., 2021) we analyzed the weather extremes on the summits of Everest and K2 in the climbing and the winter seasons using monthly means between 1979 and 2019.

All 8K peaks have already been ascended in the climbing and the winter seasons, so here we analyze and summarize weather conditions that mountaineers have experienced in the most extreme climbs of 8K peaks to determine the environmental high-altitude extremes of human survival. We evaluate the Death Zone’s weather extremes in all ascents of Everest and K2 without oxygen, as well as in all winter ascents of 8K peaks in the Himalayas and Karakoram with and without supplemental oxygen. By broadening the knowledge of the most extreme survivable environmental conditions expedition leaders, physiologists and physicians will have the facts they need to prepare climbers for extreme high-altitude weather and to maximize mountaineers’ chances of reaching summits while minimizing the risks of their attempts.

## Materials and Methods

### Materials

To survey all ascents in the most extreme environmental conditions, our analyses included all successful ascents without oxygen of the two highest 8K peaks: Mount Everest (8848 m) and K2 (8611 m) in the two the highest mountain ranges, the Himalayas and Karakoram. We also examined all successful winter ascents with and without supplemental oxygen of all the other 8K peaks. Our analyses covered the period from the first ascents of Everest and K2 without oxygen in 1978 to the first winter ascent of K2 in 2021.

We analyzed climatic conditions climbers have endured on the summits of 8K peaks, so we used a successful ascent as the unit of our analysis. We considered an attempt successful if a climber reached the summit, whether the climber returned to base camp or died during the descent. We excluded attempts that terminated before reaching the summit, whatever the reason. Each successful ascent was a separate data point. Winter-season ascents included the meteorological winter (December–February) and the calendar winter season from December 22 to March 20. We obtained the number, dates and times of ascents of Himalayan 8K peaks from the Himalayan Database (Salisbury and Hawley, 2020) and ascents of Karakoram’s 8K peaks from mountaineering journals, books and online sources (8000ers, 2021).

We identified 528 ascents that met our criteria: 499 without oxygen (423 non-winter and 76 in winter) and 29 oxygen-supported winter ascents. Of the 423 non-winter ascents, 210 were of Everest (ME-noO_2_) and 213 were of K2 (K2-noO_2_). Most non-winter ascents of Everest without oxygen (93%, 196/210) and K2 (88%, 188/213) were accomplished in the climbing season, May and October for Everest (Salisbury and Hawley, 2020; Matthews et al., 2020b), and July and August for K2 (8000ers, 2021).

The altitude and the latitude of the summit determine its weather, so we divided the 76 winter ascents of 8K peaks without oxygen into five groups for our analysis: 1 of Everest (wME-noO_2_), 1 of K2 (wK2-noO_2_), 10 of high Himalayan 8K peaks (wH&H-noO_2_), 50 of low Himalayan 8K peaks (wL&H-noO_2_) and 14 of low Karakoram 8K peaks (wL&K-noO_2_) (Table 1).

**TABLE 1:** Characteristics of ascents of 8000 m peaks.

Ascents group	Number of ascents	Ascent altitude [m] ± SD	Summit days	Climbers’ origin (highlander/lowlander)	Sex (female/male)	Climbers’ Age ± SD (min-max)	Deaths on descent
ME-noO_2_	210	8848	111	63/147	7/203	35 ± 6 (20-55)	13 (6%)
K2-noO_2_	213	8611	59	17/196	10/203	38 ± 7 (22-60)	26 (12%)
wME-noO_2_	1	8848	1	1/0	0/1	39	0
wK2-noO_2_	1	8611	1	1/0	0/1	37	0
wH&H-noO_2_	10	8504 ± 43	4	4/6	0/10	38 ± 4 (30-45)	0
wL&H-noO_2_	50	8158 ± 42	19	3/47	1/49	32 ± 5 (22-47)	2 (4%)
wL&K-noO_2_	14	8077 ± 38	5	1/13	1/13	39 ± 8 (28-58)	3 (21%)
w8000-yesO_2_	29	8677 ± 192	11	13/16	0/29	32 ± 5 (26-46)	4 (14%)
wME-yesO_2_	14	8848	7	1/14	0/14	32 ± 5 (26-45)	1 (7%)
wK2-yesO_2_	9	8611	1	9/0	0/9	35 ± 6 (31-46)	0

*ME-noO_2_, Everest non-oxygen non-winter ascents; K2-noO_2_, K2 non-oxygen non-winter ascents; wME-noO_2_, Everest non-oxygen winter ascents; wK2-noO_2_, K2 non-oxygen winter ascents; wH&H-noO_2_, high Himalayan 8K peaks non-oxygen winter ascents; wL&H-noO_2_, low Himalayan 8K peaks non-oxygen winter ascents; wL&K-noO_2_, low Karakoram 8K peaks non-oxygen winter ascents; w8000-yesO_2_, 8K peaks oxygen-supported winter ascents; wME-yesO_2_, Everest winter oxygen-supported ascents; wK2-yesO_2_, K2 winter oxygen-supported ascents.*

The wH&H-noO_2_ ascents included winter climbs of four Himalayan 8K peaks higher than 8450 m: Kangchenjunga (8586 m), Kangchenjunga South (8494 m), Lhotse (8516 m) and Makalu (8481 m). The group of wL&H-noO_2_ ascents included winter climbs of five Himalayan 8K peaks lower than 8250 m: Cho Oyu (8201 m), Dhaulagiri (8167 m), Manaslu (8156 m), Annapurna (8091 m) and Shisha Pangma (8013 m). The wL&K-noO_2_ ascents included winter climbs of four Karakoram 8K peaks lower than 8250 m: Nanga Parbat (8126 m), Gasherbrum I (8068 m), Broad Peak (8051 m) and Gasherbrum II (8035 m). Nanga Parbat is a Himalayan peak but at Karakoram’s latitude, so we included it in the wL&K-noO_2_ group. K2 is the only high 8K peak in the Karakoram range, so we analyzed it separately as wK2-noO_2_ (Table 1).

Of the 29 oxygen-supported ascents of 8K peaks in winter (w8000-yesO_2_) 14 were of Everest (wME-yesO_2_), 9 were of K2 (wK2-yesO_2_), 4 were of high Himalayan peaks and 2 were of low Himalayan peaks (Table 1).

The 528 ascents we analyzed were accomplished over 211 days on the summits. Of these ascents 509 were by male climbers and 19 by females. Their average age was 36 ± 7 years. There were 425 ascents by lowlanders and 103 by highlanders. We included Tibetans and populations with Tibetan ancestry in the highlander group: Nepali highlanders (Sherpa, Rai, Magar, Tamang) (Cole et al., 2017) and Pakistani highlanders (Balti) (Yang et al., 2020). Forty-eight climbers died on their descents (Table 1).

### Methods

These 8K peaks lack weather stations that can provide *in situ* data, so we used the ERA5 Reanalysis as our primary source of meteorological conditions. ERA5 is a state-of-the-art database provided by the European Centre for Medium-Range Weather Forecasts (ECMWF) (Hersbach et al., 2020). This database assimilates all available observations, including satellite data, and is provided at the high spatial resolution of 0.25 × 0.25 degrees of latitude and longitude. The calculated values of weather conditions at the summit altitude should be interpreted as averages for the roughly rectangular area defined by the reanalysis model’s resolution for the location of the mountain. The rectangles have sides of about 28 km by 24 km in the Himalayas and 28 km by 22 km at the latitude of Karakoram.

We analyzed the climatic factors that most limit human performance and survival at high altitude: BP, Temp and Wind. Our research examined four meteorological variables at the 300, 350, and 400 hPa isobaric levels. We used hourly values of geopotential height, air temperature and wind vector components (u, zonal; v, meridional) at these three standard synoptic levels to calculate BP, Temp and Wind at the desired height. When the exact time of the ascent was unavailable, we estimated the environmental conditions using a Gaussian weighted average for the day of the successful climb: 11:30 Nepal Time (NPT) ± 3.5 h for Everest ascents, 15:30 Pakistan Time (PKT) ± 3.3 h for K2 ascents and 13:30 NPT ± 2.5 h for winter ascents of 8K peaks. The summiting hour was unavailable for 10 ascents of Everest (5%), 25 ascents of K2 (12%) and 9 winter ascents of Himalayan 8K peaks without oxygen (12%).

We had to calculate BP at the summit levels of the selected 8K peaks before interpolating Temp and Wind. We calculated BP by fitting a non-linear regression model to each instance of the air pressure-height profile. Calibrated model coefficients were then used to estimate BP at a given height. This provided the data we needed to estimate the values of other variables. Temp and Wind were derived from linear interpolation of values at predefined isobaric surfaces from ERA5. Temperature lapse rates change with season (Dillon et al., 2006), so Temp was calculated individually for each instance.

From the Temp and Wind results we estimated the WCT and FFT thermal stress indices. Our WCT and FFT calculations we based on formulas by Osczevski and Bluestein and by Tikuisis and Osczevski (Tikuisis and Osczevski, 2003; Osczevski and Bluestein, 2005; Moore and Semple, 2011):

$$\mathrm{WCT}{[}^{\circ }C]=13.12+0.621T-11.37V^{0.16}+0.3965TV^{0.16}$$

$$\mathrm{FFT}\,\left[\min\right]=\left[-24.5\,\left(0.667\,V+4.8\right)+2111\right]\,{\left(-4.8-T\right)}^{-1.668}$$

where V is the wind speed (km⋅h^–1^) 10 m above the surface and T is the air temperature (°C).

### Statistical Analysis

We used Statistica 13.1 (StatSoft, United States) for our calculations, testing the normality of data with the Shapiro-Wilks W-test, then the homogeneity of variance with the Brown-Forsythe test. To assess the statistical significance of differences we used analysis of variance and Tukey’s *post hoc* test. Our results are expressed as means with a standard deviation (x ± SD). We measured the relative variability of all parameters using a coefficient of variation (CV) and analyzed any associations of the parameters using Pearson’s correlation coefficient (r). We set statistical significance at *p* < 0.05 for all our analyses.

## Results

### Barometric Pressure

**FIGURE 1:**
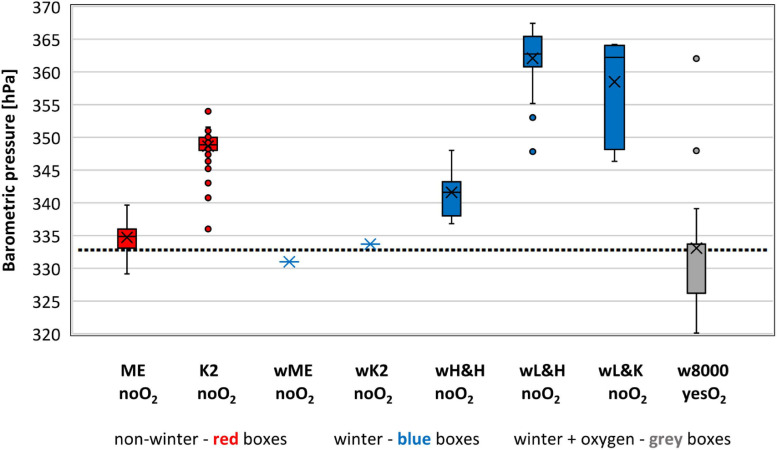
Barometric pressure experienced on the summits we analyzed. Sample size – 499 ascents without oxygen support and 29 with oxygen support (1978–2021). Bars extend to the maximum and minimum BP, boxes span the 25th–75th percentiles, crosses inside the boxes mark mean values, horizontal lines inside the boxes represents median values, dots mark outliers, dotted line represents 10th percentile value of 333 hPa for all 499 ascents with no supplemental oxygen.

Of all 499 ascents of 8K peaks without oxygen that we analyzed, 50 experienced BP ≤ 333 hPa (10th percentile), all on Everest (Figure 1 and Table 2). The average BP of 335 ± 2 hPa in non-winter ascents of Everest without oxygen was significantly lower (p < 0.01) than in the other groups of oxygen-unaided ascents except for one winter ascent of Everest and one winter ascent of K2 (Table 3). BP of 329 hPa was the lowest endured without oxygen support and was experienced in two ascents of Everest in April (Table 4).

**TABLE 2:** Number of ascents under the most severe environmental conditions of barometric pressure, ambient temperature, wind chill temperature and facial frostbite time within the 10th percentile and wind speed within 90th percentile for ascents without supplemental oxygen.

Ascents group and number	BP ≤ 333 hPa	Temp ≤ −30°C	Wind ≥ 25 m⋅s^–1^	WCT ≤ −54°C	FFT ≤ 3 min
ME-noO_2_ (210)	49 (23%)	12 (6%)	5 (2%)	3 (1%)	3 (1%)
K2-noO_2_ (213)	0	2 (1%)	4 (2%)	2 (1%)	2 (1%)
wME-noO_2_ (1)	1 (100%)	1 (100%)	1 (100%)	1 (100%)	1 (100%)
wK2-noO_2_ (1)	0	1 (100%)	0	1 (100%)	0
wH&H-noO_2_ (10)	0	6 (60%)	9 (90%)	8 (80%)	10 (100%)
wL&H-noO_2_ (50)	0	14 (28%)	31 (62%)	25 (50%)	33 (66%)
wL&K-noO_2_ (14)	0	14 (100%)	0	10 (71%)	1 (7%)
w8000-yesO_2_ (29)	14 (48%)	28 (97%)	17 (59%)	28 (97%)	19 (66%)
wME-yesO_2_ (14)	14 (100%)	14 (100%)	12 (86%)	14 (100%)	14 (100%)
wK2-yesO_2_ (9)	0	9 (100%)	0	9 (100%)	0

*ME-noO_2_, Everest non-oxygen non-winter ascents; K2-noO_2_, K2 non-oxygen non-winter ascents; wME-noO_2_, Everest non-oxygen winter ascents; wK2-noO_2_, K2 non-oxygen winter ascents; wH&H-noO_2_, high Himalayan 8K peaks non-oxygen winter ascents; wL&H-noO_2_, low Himalayan 8K peaks non-oxygen winter ascents; wL&K-noO_2_, low Karakoram 8K peaks non-oxygen winter ascents; w8000-yesO_2_, 8K peaks oxygen-supported winter ascents; wME-yesO_2_, Everest winter oxygen-supported ascents; wK2-yesO_2_, K2 winter oxygen-supported ascents.*

*BP, barometric pressure; Temp, temperature; Wind, wind speed; WCT, wind chill equivalent temperature; FFT, facial frostbite time.*

**TABLE 3:** Barometric pressure, ambient temperature, wind speed and thermal stress indices: WCT and FFT for the 499 ascents without oxygen support and 29 oxygen-supported ascents of 8K peaks we analyzed: number of ascents, means ± standard deviation (SD), coefficient of variation (CV%), minimum and maximum values.

Ascent group and number	BP [hPa]	Temp [°C]	Wind [m⋅s^–1^]	WCT [°C]	FFT [min]
ME-noO_2_ (210)	335 ± 2 (1%) min 329, max 340	−25 ± 3 (12%) min −33, max −19	12 ± 5 (43%) min 2, max 27	−40 ± 5 (13%) min −57, max −26	9 ± 4 (39%) min 2, max 22
K2-noO_2_ (213)	349 ± 2 (1%) min 336, max 354	−19 ± 3 (15%) min −36, max −14	12 ± 5 (40%) min 1, max 26	−33 ± 5(16%) min −60, max -19	17 ± 7 (39%) min 2, max 38
wME-noO_2_ (1)	331	−33	26	−59	2
wK2-noO_2_ (1)	334	−40	5	−55	5
wH&H-noO_2_ (10)	342 ± 4 (1%) min 337, max 348	−32 ± 4 (13%) min −37, max −27	41 ± 9 (21%) min 22, max 48	−60 ± 6 (10%) min −65, max −51	−2 ± 2 (109%) min −4, max 2
wL&H-noO_2_ (50)	362 ± 5 (1%) min 348, max 367	−29 ± 4 (14%) min −40, max −25	26 ± 10 (38%) min 12, max 46	−52 ± 6 (11%) min −64, max −41	2 ± 3 (141%) min −4, max 9
wL&K-noO_2_ (14)	358 ± 7 (2%) min 346, max 364	−38 ± 5 (12%) min −45, max −34	12 ± 4 (35%) min 7, max 18	−58 ± 6 (11%) min −67, max -51	4 ± 1 (24%) min 3, max 5
w8000-yesO_2_ (29)	333 ± 10 (3%) min 320, max 362	−36 ± 4 (11%) min −41, max −27	24 ± 14 (59%) min 5, max 46	−60 ± 6 (10%) min −69, max −47	2 ± 3 (166%) min -3, max 5
wME-yesO_2_ (14)	326 ± 3 (1%) min 320, max 331	−36 ± 4 (10%) min −41, max −31	36 ± 7 (18%) min 24, max 46	−64 ± 5 (7%) min −69, max -57	−1 ± 1 (275%) min −3, max 2
wK2-yesO_2_ (9)	334	−40	5	−55	5

*ME-noO_2_, Everest non-oxygen non-winter ascents; K2-noO_2_, K2 non-oxygen non-winter ascents; wME-noO_2_, Everest non-oxygen winter ascents; wK2-noO_2_, K2 non-oxygen winter ascents; wH&H-noO_2_, high Himalayan 8K peaks non-oxygen winter ascents; wL&H-noO_2_, low Himalayan 8K peaks non-oxygen winter ascents; wL&K-noO_2_, low Karakoram 8K peaks non-oxygen winter ascents; w8000-yesO_2_, 8K peaks oxygen-supported winter ascents; wME-yesO_2_, Everest winter oxygen-supported ascents; wK2-yesO_2_, K2 winter oxygen-supported ascents.*

*BP, barometric pressure; Temp, temperature; Wind, wind speed; WCT, wind chill equivalent temperature; FFT, facial frostbite time.*

**TABLE 4:** Mountaineers’ survival records.

	Ascent group	Date	Names	BP [hPa]	Temp [°C]	Wind [ms^–^^1^]	WCT [°C]	FFT [min]
1	ME-noO_2_	1985.04.29	Ang Rita Sherpa	**329**	−33	7	−48	6
2	ME-noO_2_	1984.04.20	Christo Ivanov Prodanov^†^	**329**	−31	21	−54	3
3	wL&K-noO_2_ (NP)	2018.01.25	T. Mackiewicz^†^, E. Revol	346	**−45**	10	**-67**	3
4	wL&K-noO_2_ (GI)	2012.03.09	A. Bielecki, J. Goła̧b	348	**−44**	10	**−66**	3
5	wH&H-noO_2_ (KG)	1986.01.11	K. Wielicki, J. Kukuczka	337	−28	**48**	−56	**−4**
6	wL&H-noO_2_ (SP)	2005.01.14	P. Morawski, S. Moro	366	−28	**46**	−55	**−4**
7	wH&H-noO_2_ (KS)	2012.02.15	P. Kunz, K. Chhiri Lama, L. Ongya Sherpa, N. Wangdi Sherpa, T. Dorje Sherpa	342	−35	**46**	−65	−3
8	wME-noO_2_	1987.12.22	Ang Rita Sherpa	331	−33	26	−59	2
9	ME-noO_2_	1985.10.30	N. Yamada	331	−32	27	−57	2
10	K2-noO_2_	2021.01.16	N. Purja	334	−40	5	−55	5
11	K2-noO_2_	2007.10.02	D. Urubko, S. Samoilov	336	−36	22	−60	2
12	wH&H-noO_2_ (LH)	1988.12.31	K. Wielicki	338	−37	22	−63	2
13	wME-yesO_2_	1980.02.17	L. Cichy, K. Wielicki	320	−41	24	−69	1
14	wME-yesO_2_	1982.12.27	Y. Kato^†^	328	−31	46	−59	−3

*Numbers in bold represent the record value.*

*Mountaineer survival records without supplemental oxygen:*

*Barometric pressure (BP) of 329 hPa: 1, 2.*

*Air temperature (Temp) ≤ −44°C: 3, 4.*

*Wind speed (Wind) ≥ 46 m⋅s^–1^: 5, 6, 7.*

*Wind chill temperature (WCT) ≤ −66°C: 3, 4.*

*Facial frostbite time (FFT) of -4 min: 5, 6.*

*Combined conditions: BP ≤ 333 hPa, Temp ≤ –30°C, Wind ≥ 25 m⋅s^–1^, WCT ≤ –54°C and FFF ≤ 3 min: 8, 9.*

*Combined conditions: BP ≤ 346 hPa, WCT ≤ –54°C: 1, 2, 3, 5, 7, 8, 9, 10, 11, 12.*

*Mountaineer survival records with supplemental oxygen: the most extreme combined conditions: 13, 14.*

*ME-noO_2_, Everest non-oxygen non-winter ascents; K2-noO_2_, K2 non-oxygen non-winter ascents; wME-noO_2_, Everest non-oxygen winter ascents; wK2-noO_2_, K2 non-oxygen winter ascents; wH&H-noO_2_, high Himalayan 8K peaks non-oxygen winter ascents; wL&H-noO_2_, low Himalayan 8K peaks non-oxygen winter ascents; wL&K-noO_2_, low Karakoram 8K peaks non-oxygen winter ascents; w8000-yesO_2_, 8K peaks oxygen-supported winter ascents; wME-yesO_2_, Everest winter oxygen-supported ascents; NG, Nanga Parbat, GI, Gasherbrum I, KG, Kangchenjunga, SP, Shisha Pangma, KS, Kangchenjunga South, LH, Lhotse, ^†^died during descent; BP, barometric pressure; Temp, temperature; Wind, wind speed; WCT, wind chill equivalent temperature; FFT, facial frostbite time.*

Of 29 oxygen-supported ascents of 8K peaks in winter, all 14 ascents of Everest encountered BP ≤ 333 hPa (average of 326 ± 3 hPa); 12 encountered BP < 329 hPa, the lowest recorded for ascents without oxygen support (Figure 1 and Tables 2, 3). The lowest BP recorded was 320 hPa for an ascent with supplemental oxygen (Table 4).

Of the 50 ascents of Everest without oxygen experiencing BP ≤ 333 hPa, 23 were by highlanders and 27 by lowlanders, 36% and 18%, respectively, of all ascents without oxygen of Everest by those groups. Highlanders endured lower BP of 334 ± 2 hPa in these climbs than lowlanders at 335 ± 2 hPa (*p* < 0.01). Ang Rita Sherpa endured BP ≤ 333 hPa in 4 of his 9 ascents of Everest without oxygen, including the one in winter.

The highest average BP of 349 ± 2 hPa was encountered in non-winter ascents of K2 without oxygen, 14 hPa higher than in non-winter ascents of Everest without oxygen (Figure 1 and Table 3).

### Air Temperature

**FIGURE 2:**
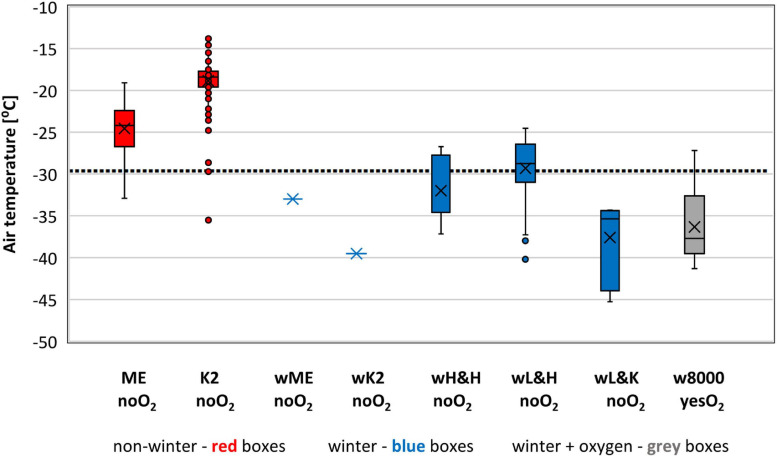
Air temperature experienced on the summits we analyzed. Sample size – 499 ascents without supplemental oxygen and 29 with supplemental oxygen (1978–2021). Bars extend to the maximum and minimum BP, boxes span the 25th–75th percentiles, crosses inside the boxes mark mean values, horizontal lines inside the boxes represents median values, dots mark outliers, dotted line represents 10th percentile value of – 30°C for all 499 ascents without supplemental oxygen.

Air temperatures ≤−30°C (10th percentile) were experienced in 50 of the 499 ascents of 8K peaks without oxygen that we analyzed: 36 in winter. Half of the non-winter ascents with the lowest Temp were undertaken outside the typical climbing season (Everest – 4 in April, 1 in the latest-ever October ascent; K2 – 2 in October), (Figure 2 and Table 2). The lowest average Temp of −38 ± 5°C was recorded in winter ascents of low Karakoram 8K peaks (wL&K-noO_2_) (Table 3). This was significantly lower (*p* < 0.01) than the average Temp in all the other groups of ascents without oxygen except one winter ascent of K2 (Table 3). The lowest Temp of ≤−44°C was endured by four climbers in winter ascents of Nanga Parbat and Gasherbrum I, two low 8K Karakoram peaks (Table 4).

The average Temp of −36 ± 4°C in 29 oxygen-supported 8K ascents in winter did not differ statistically from the mean Temp in wL&K-noO_2_ ascents (Table 3). Almost all oxygen-aided winter ascents (97%) were accomplished in Temp of ≤−30°C (Figure 2 and Table 2). The lowest Temp of −41°C was experienced in the first oxygen-aided ascent of Everest in winter (Table 4).

The highest average Temp of −19 ± 3°C was recorded in non-winter ascents of K2 without oxygen. This was 6°C higher than in non-winter ascents of Everest without oxygen (*p* < 0.01) (Figure 2 and Table 3).

### Wind Speed

**FIGURE 3:**
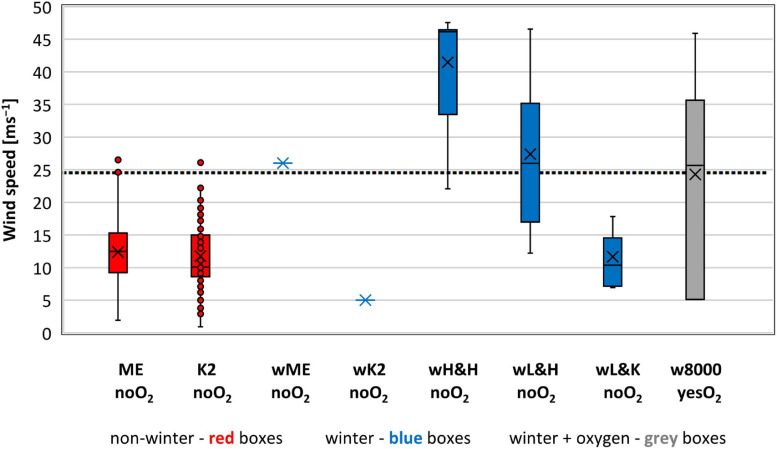
Wind speed experienced on the summits we analyzed. Sample size – 499 ascents without supplemental oxygen and 29 with supplemental oxygen (1978–2021). Bars extend to the maximum and minimum wind speed, boxes span the 25th–75th percentiles, crosses inside the boxes mark mean values, horizontal lines inside the boxes represents median values, dots mark outliers, dotted line represents 90th percentile value of 25 m⋅s^–1^ for all 499 ascents without supplemental oxygen.

Fifty of the 499 ascents without oxygen (90th percentile) that we analyzed encountered Wind ≥ 25 m⋅s^–1^ (Figure 3 and Table 2), 41 (82%) in winter ascents of 8K Himalayan peaks. Of the 9 non-winter ascents with Wind ≥ 25 m⋅s^–1,^ 5 were of Everest (4 in October, 1 in May) and 4 of K2 (the earliest four summer ascents on one summit day, 13 June). The highest average Wind of 41 ± 9 m⋅s^–1^ was recorded in winter ascents without oxygen of high Himalayan 8K peaks (wH&H-noO_2_) (Table 3). This Wind was significantly higher (*p* < 0.01) than all other groups of oxygen-unaided ascents that we analyzed (Table 3). The mean Wind value in winter ascents of low Himalayan 8K peaks (wL&H-noO_2_) was twice as high as encountered in corresponding winter ascents of low Karakoram 8K peaks (wL&K-noO_2_) (*p* < 0.01) (Table 3). Nine climbers experienced the highest Wind of ≥ 46 m⋅s**^–^**^1^ in winter ascents of Kangchenjunga, Kangchenjunga South and Shisha Pangma (Table 4). Significantly higher (*p* < 0.01) Wind of 17 ± 7 m⋅s**^–^**^1^ was encountered in all 13 non-winter ascents by lowlanders of Everest without oxygen that ended with deaths on their descents compared with an average Wind of 12 ± 5 m⋅s^–1^ in 134 ascents by lowlanders that ended safely.

Of the 29 oxygen-supported 8K ascents in winter that we analyzed, 85% in the Himalayas were climbed with Wind ≥25 m⋅s^–1^. The highest Wind of 46 m⋅s^–1^ was experienced in a winter ascent of Everest with oxygen (Table 4).

The lowest Wind was recorded in non-winter ascents without oxygen of Everest (12 ± 5 m⋅s^–1^) and of K2 (12 ± 5 m⋅s^–1^) (*p* > 0.5) (Figure 3 and Table 3).

### Wind Chill Temperature

Wind chill temperature ≤−54°C (10th percentile) was experienced in 50 of the 499 ascents of 8K peaks without oxygen that we analyzed. Most (90%) of the 50 ascents with the lowest WCT were in winter (Table 2). All five non-winter ascents encountering a WCT of ≤−54°C were accomplished out of the typical climbing season (Everest – 2 in April, 1 – the latest October ascent; K2 – 2 in October). The lowest average WCT of −60 ± 6°C and −58 ± 6°C were recorded in winter ascents of high Himalayan 8K peaks (wH&H-noO_2_) and low Karakoram 8K peaks (wL&K-noO_2_) (Table 3). Significantly higher average WCT (*p* < 0.01) was recorded in other groups of ascents without oxygen, except one winter ascent of Everest (Table 3). The lowest WCT of ≤−66°C was endured by four climbers in winter ascents of Nanga Parbat and Gasherbrum I (Table 4).

The average WCT of −66 ± 6°C in 29 oxygen-supported winter ascents of 8K peaks did not differ statistically from the mean WCT for wH&H-noO_2_ climbs (Table 3). All but 1 winter ascent with oxygen were accomplished in WCT ≤ −54°C (Table 2). The lowest WCT of −69°C was experienced in the first oxygen-aided ascent of Everest in winter (Table 4).

The highest average WCT of −33 ± 5°C was recorded in non-winter ascents of K2 without oxygen. This was 7°C higher than in non-winter ascents of Everest without oxygen (*p* < 0.001) (Table 3).

### Facial Frostbite Time

We noted 50 ascents (10th percentile) with an FFT ≤ 3 min among all 499 ascents without oxygen that we analyzed (Table 2). Most (90%) of these 50 ascents were in winter (Table 2). Three of five non-winter ascents with an FFT ≤ 3 min were undertaken outside the typical climbing season: Everest – 1, the latest October ascent; K2 – 2 in October, (Table 2). The lowest average FFT of -2 ± 2 min was recorded in wH&H-noO_2_ ascents (Table 3). This was significantly shorter (*p* < 0.01) than in non-winter ascents of Everest and K2, and not statistically different from other oxygen-unaided winter ascents (Table 3). The shortest FFT of -4 min was experienced by four climbers in winter ascents of Kangchenjunga and Shisha Pangma (Table 4).

Of 29 oxygen-supported ascents of 8K peaks in winter, all Himalayan climbs except 1 were accomplished in FFT ≤ 3 min. The shortest FFT of -3 min was experienced in an oxygen-supported ascent of Everest in winter (Table 4).

The longest average FFT of 17 ± 7 min was recorded in non-winter ascents of K2 without oxygen, 8 min longer than for non-winter ascents of Everest without oxygen (*p* < 0.001) (Table 3).

### Combined Weather Conditions

**FIGURE 4:**
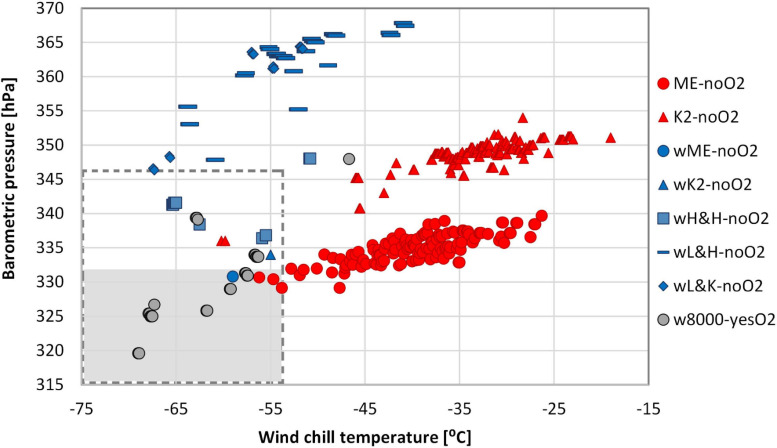
Combined barometric pressure (BP) and wind chill temperature (WCT). Sample size – 499 ascents without supplemental oxygen and 29 with oxygen support (1978–2021). Dashed line box covers ascents in combined conditions of BP ≤ 346 hPa and WCT ≤ −54°C, gray area represents ascents in combined conditions of BP ≤ 333 hPa and WCT ≤ −54°C.

Seventeen (3%) of the 499 ascents without oxygen that we analyzed were accomplished in combined conditions of BP ≤ 346 hPa (median) and WCT ≤ −54°C (10th percentile (Figure 4). Unaided assents included 4 of Everest (1 – winter, 3 – non-winter), 3 of K2 (1 – winter, 2 –non-winter), 2 of wL&K-noO_2_ and 8 of wH&H-noO_2_ (Table 4, Figure 4). Of the 29 oxygen-supported ascents of 8K peaks most (26, 90%) were in these extreme conditions (Figure 4). Only 4 ascents of the 499 we analyzed were in combined conditions of BP ≤ 333 hPa and WCT ≤ −54°C, the 10th percentile for 8K ascents without oxygen. All 4 were on Everest (1 – winter, 3 out of regular season: 1 – late October, 2 – April).

Only 2 of the 499 ascents of 8K peaks were accomplished in the combined 10th percentile for all the conditions we calculated (BP ≤ 333 hPa, Temp ≤ −30°C, Wind ≥ 25 m⋅s^–1^, WCT ≤ −54°C and FFT ≤ 3 min). Both climbs were the latest in the year of all ascents of Everest without oxygen (30 October and 22 December) (Table 4). Of 29 oxygen-supported ascents of 8K peaks in winter, 12 of 14 ascents of Everest were in these most extreme combined conditions (Figure 4).

We found a strong positive correlation of BP and Temp (*r* ≥ 0.7; *p* < 0.05) in most of the ascents we analyzed: non-winter ascents of Everest and K2, winter ascents of low Karakoram and Himalayan 8K peaks, and oxygen-supported ascents of Everest in winter. We observed a fairly negative correlation of BP and Wind (*r* ≤ -0.3; *p* < 0.05) and a poor negative correlation of Temp and Wind (*r* ≤ -0.1; *p* < 0.05) in non-winter ascents of Everest and K2. There was no significant correlation between BP and Wind in all winter-ascent groups, and of Temp and Wind in most of the winter groups. Temp and Wind were linked only in winter ascents of Everest with oxygen, where we noted a moderate positive correlation (*r* = 0.7; *p* < 0.05).

## Discussion

Low BP, low Temp and high Wind are the main environmental factors that limit human performance and survival at high altitude (West and Wagner, 1980; West et al., 1983a, 2007b; Huey et al., 2001; Huey and Eguskitza, 2001; Moore and Semple, 2012; Moore et al., 2012). We aimed to evaluate weather extremes in the Death Zone and to determine the most severe environmental conditions that mountaineers have experienced. No *in situ* data were available for the summits and dates we analyzed, so we derived our calculations from ERA5, a state-of-the-art meteorological database. Previously published assessments of weather on the 8K peaks were calculated from far less precise radiosonde data (West et al., 1983b) and from US National Centers for Environmental Prediction (NCEP) Reanalysis data (Moore and Semple, 2011). The NCEP Reanalysis is one of the earliest meteorological datasets with a 10-times-lower spatial resolution of 2.5 × 2.5 degrees of latitude and longitude (Kalnay et al., 1996) compared with the 0.25 × 0.25 degree resolution of the ERA5 dataset (Hersbach et al., 2020). Everest’s summit BP calculated from ERA5 and corrected with *in situ* measurements from automatic weather stations installed on the mountain’s South Col (7945 m) and Balcony (8430 m) as described by Matthews et al. (2020b) differed by about 1 hPa from results based on ERA5 alone as reported in our earlier article (Szymczak et al., 2021).

### Barometric Pressure

Our finding that the lowest BP was experienced in winter ascents of Everest accorded with previous observations that higher altitudes and colder seasons lead to lower BP (Brunt, 1952; West et al., 1983b, 2007c; West, 1996; Whiteman, 2000). The most extreme mean BP of 326 ± 3 hPa that we recorded was experienced in oxygen-aided ascents of Everest in winter. Most (86%) of those ascents were in December, so the similarity of the calculated mean BP to Everest summit’s average December values of 326 hPa (Matthews et al., 2020b) and 327 hPa (Szymczak et al., 2021) could be expected. However, the lowest BP recorded in our study of 320 hPa, experienced with oxygen support on the summit of Everest in February, was below the summit’s previously reported February mean of 323 hPa (Matthews et al., 2020b) and 324 hPa (Szymczak et al., 2021). The lowest BP of 334 hPa experienced on K2’s summit in January was also far from its average in January of 325 hPa (Szymczak et al., 2021). Similarly the BP of 331 hPa experienced in the only ascent of Everest without oxygen in December was higher than the peak’s December’s average. The difference we noted between the BP experienced by successful climbers and the monthly midwinter mean BP presented in other works (Matthews et al., 2020b; Szymczak et al., 2021) corresponded with the high daily variability recorded for BP in winter on Everest (Matthews et al., 2020b). Therefore the routine use of barometric pressure forecasts, as suggested by Moore and Semple (2011, 2012), would enable climbers to choose the most favorable summit-bid BP window and should be implemented, especially for winter expeditions.

Supplemental use of oxygen reduces the risk of high-altitude deterioration and the death of mountaineers at extreme altitudes (West, 1998; Huey and Eguskitza, 2000, 2001; Huey et al., 2001). One in 34 climbers using supplemental oxygen dies in descents from Everest compared with one in 12 without oxygen support on Everest and one in five on K2 (Huey and Eguskitza, 2000). Our result showing that BP of 320 hPa, the lowest for oxygen-aided ascents, was significantly lower than the BP of 329 hPa for climbs without oxygen, strongly suggests that oxygen use enables survival of extremely low BP. A BP of 315 hPa was the lowest a human has ever survived without supplemental oxygen. It was experienced for about 20 min in a hypobaric chamber by four volunteers in the Operation Everest I study (Houston and Riley, 1947) where the BP at Everest’s summit was overestimated by using a simplified formula for aviation standard atmosphere (ICAO, 1964). Considering that humans can therefore survive a BP of 315 hPa, and that Everest and K2’s average midwinter BP is below 326 hPa (Matthews et al., 2020b; Szymczak et al., 2021) at the summits, with the rising interest in winter climbing at extreme altitudes (Benavides, 2021a) we expect that a new BP survival record will be established in future winter expeditions without oxygen on those peaks.

### Thermal Stress

We noted that the most severe Temp in the Death Zone was experienced in winter ascents of 8K peaks in the Karakoram range. Latitude, altitude, and season mainly determine air temperature (Whiteman, 2000). Our results showed that the average Temp in winter ascents of low Karakoram 8K peaks was lower than in winter ascents of low Himalayan 8K peaks as well as high Himalayan 8K peaks that are 400 m higher. This result suggests that in winter Karakoram’s latitude 8° further north has a greater effect on Temp than the 400 m altitude difference between 8K peaks. This result accords with the lower average midwinter Temp on K2’s summit, the highest in the Karakoram range, than on the 237 m higher Everest further south (Szymczak et al., 2021).

The highest Wind recorded in our study occurred in winter Himalayan ascents. Wind was significantly more severe in winter ascents in the Himalayas than on Karakoram. Our calculation tallies with trends of the global jet stream analyzed by other authors (Moore and Semple, 2004; Archer and Caldeira, 2008; Pena-Ortiz et al., 2013) and the higher average midwinter Wind recorded on Everest than on K2 (Szymczak et al., 2021). The Northern Hemisphere jet stream splits over the Atlantic Ocean into the Subtropical jet stream (STJ) and the Polar Front jet stream (PFJ) (Pena-Ortiz et al., 2013). At the longitude of the Himalayas and Karakoram the STJ flows between 20° N and 35° N, above the Himalayas; the PFJ flows between 40° N and 70° N (Pena-Ortiz et al., 2013). This leaves a 5° gap between 35° N and 40° N that lies over Karakoram, which would explain the significantly lower Wind in winter ascents of Karakoram 8K peaks compared with the Himalayas.

We estimated Wind at the altitude of 8K peaks, where there is no natural wind protection by rock formations or slopes. However, wind direction and mountain topography should be considered when wind exposure is analyzed for different routes up a mountain. A route on the windward side of a mountain will expose a climber to more extreme Wind than a route on the leeward side. Jet streams are westerly winds (Pena-Ortiz et al., 2013), therefore routes on the east walls of 8K peaks are less exposed to Wind, which should be considered by winter climbers.

The combined effects of Temp and Wind can be gauged with thermal stress indices such as WCT (Osczevski and Bluestein, 2005) and FFT (Tikuisis and Osczevski, 2003). Our results show that the lowest WCT and the shortest FFT were encountered in winter ascents of 8K peaks. Our results for WCT and FFT in ascents of Everest were similar to the WCT of −45°C and the FFT of 7 min in summer and the WCT of −65°C and the FFT < 1 min in winter assessed with the NCEP Reanalysis (Moore and Semple, 2011). Wind chill temperature depends more on ambient temperature than on wind (Osczevski and Bluestein, 2005), so the extremely low temperatures of Karakoram’s Death Zone in winter strongly influenced its WCT. The WCT index is limited to wind speeds below 27 m⋅s^–1^ (Osczevski and Bluestein, 2005), so the effect of extremely high Wind in the Himalayan winter is likely underestimated. The skin’s rate of cooling is more sensitive to wind speed than to air temperature (Tikuisis and Osczevski, 2003), so higher winter Wind in the Himalayas explained the lowest average FFT that we estimated for the highest 8K peaks in this range. FFTs below 0 min signal that the extreme combined Temp and Wind in the Death Zone in winter might fall beyond the scope of the FFT index. Reduced air density at high altitude decreases convective heat loss (Huey et al., 2001). Huey et al. calculated that with the 60% decline of air density from sea level to 9000 m the convective heat loss at 9000 m decreased by about 45% compared with the loss at sea level in the same conditions at a Temp of −33.5°C and wind speeds up to 28 m⋅s^–1^ (Huey et al., 2001). The standard equations for WCT and FFT (Tikuisis and Osczevski, 2003; Osczevski and Bluestein, 2005) applied in our study and others (Moore and Semple, 2011) presume the sea-level densities of air and therefore likely overestimate heat loss at altitude. Considering the estimates of Huey et al. (2001), our results for WCT and FFT should be modified to show more realistic values of heat loss at high altitude. The validation of WCT and FFT for the high-altitude low-BP environment, or a more complete human heat balance index such as the Universal Thermal Climate Index (UTCI) (Blazejczyk et al., 2012), would benefit future high-altitude research and provide a more realistic assessment of thermal stress.

### Combined Weather Extremes

Our results showed that the most extreme combined conditions of BP, Temp and Wind were experienced in winter and off-season ascents of Everest. This is explained by Everest’s extreme altitude, which determines its lowest BP, its low winter and off-season Temp, and high Wind because of the STJ’s winter activity over the Himalayas (Whiteman, 2000; Pena-Ortiz et al., 2013).

We found a strong positive correlation between BP and Temp in most of the ascents we analyzed, which agrees with similar observations by others of BP and Temp for the summits of Everest (Moore and Semple, 2011; Matthews et al., 2020b; Szymczak et al., 2021) and K2 (Szymczak et al., 2021). This also corresponds with the interrelationship of BP and Temp described by the ideal gas law (West, 1996). We noted a moderate positive correlation of Wind and Temp and no correlation of Wind and BP in winter ascents of Everest. Our results suggested that a climber in winter should expect lower Temp in a lower Wind window on Everest. Observations by Matthews et al. (2020b) that extreme low-pressure periods on Everest’s summit in winter were not associated with strong winter winds would suggest that low-pressure events might occur in low Wind windows. Therefore climbers, who usually employ wind speed to identify suitable climbing weather windows (Peplow, 2004), should also focus on barometric pressure forecasts to increase their chances of success and survival, especially in the winter season.

The interrelationship of BP and Temp with Wind was more favorable in non-winter ascents of Everest and K2. We recorded a fairly negative correlation of BP and Wind in non-winter ascents of Everest and K2, which means that in a low Wind window climbers should also expect favorably higher BP. BP’s positive correlation with Temp and negative correlation with Wind recorded for non-winter ascents of Everest and K2 supported the proposal by Moore and Semple (2011, 2012) for Everest climbers that summit BP can act as a simple and easily implementable predictor of the risk of hypothermia and frostbite. Huey et al. (2020) suggested that improved weather forecasting was one reason for the increased likelihood of summiting Everest and the lower likelihood of mountaineers dying over the past 15 years compared with 1990–2005. Therefore monitoring of Temp and BP in the base camp and at higher camps, as during the first Everest expeditions almost 100 years ago (Moore et al., 2010), should become standard practice to correct more general forecasts for high-altitude expeditions.

We found that environmental conditions in the Death Zone were more severe in non-winter ascents of Everest than of K2. BP was significantly lower on Everest, which might be explained by Everest’s 237 m higher altitude and the less favorable month of its climbing season. The highest BP and Temp on both peaks were recorded in July and August (Szymczak et al., 2021). Due to summer snowfalls caused by the monsoons (Moore, 2004; Moore and Semple, 2006, 2011), the main climbing season on Everest is mostly restricted to May (Matthews et al., 2020b; Salisbury and Hawley, 2020), which has lower BP and Temp than in July and August. The average BP in May on Everest’s summit was 6 hPa lower than in July (Matthews et al., 2020b). K2’s climbing season is in July and August (8000ers, 2021), the months with the highest BP and Temp (Szymczak et al., 2021). We found no difference in Wind for non-winter ascents of Everest and K2. Those that climb Everest and K2 in the normal season should be aware that environmental conditions in Everest’s Death Zone are more severe than K2’s. The higher death toll experienced on K2 (Huey and Eguskitza, 2000) is likely explained by the technically more difficult route to the summit or yet unexamined weather characteristics. K2’s midwinter summit BP is similar to Everest’s, but Temp and Wind are lower (Szymczak et al., 2021).

### Deaths on Descent

Our analyses of 528 ascents recorded 48 (9%) that ended with the death of a climber in the descent. Most of those deaths (26) occurred on K2 in the climbing season. Three climbers reached 8K summits in the most extreme weather conditions we recorded, but died in their descent: one climber who reached Everest’s summit with BP of 329 hPa, one climber who experienced Temp of −44°C and WCT of −67°C in winter on the summit of Nanga Parbat, and one mountaineer with oxygen support who reached Everest’s summit in winter with Wind of 46 m⋅s^–1^. We did not analyze the causes of death. Only deaths on Everest have been thoroughly analyzed (Firth et al., 2008), so further research is needed for the other 8K peaks. Firth et al. (2008) observed that severe weather is the main factor responsible for about 25% of fatalities above 7000 m on Everest. Hypothermia is responsible for 16% of all deaths on Denali (McIntosh et al., 2008). Moore and Semple (2012) presented the cases of two climbers who had to bivouac above 8500 m on descent from Everest in extreme environmental conditions: the first experienced BP of 333 hPa, Temp of −31°C and Wind of 15 m⋅s^–1^; the second experienced BP of 338 hPa, Temp −23°C, and Wind 2 m⋅s^–1^. The first climber died but the second survived. Authors have attributed this death to the higher hypoxic and hypothermic stress he experienced (Moore and Semple, 2012).

The risk of hypothermia rises to critical levels when a fatigued mountaineer stops during descent or is forced to bivouac. The lower exertion a climber requires on descent reduces heat production, which falls precipitously if the climber stops (Ainslie and Reilly, 2003). Hypothermia *per se* increases fatigue because of a reduction in muscle strength and an increase in oxygen consumption for the same intensity of exercise (Hinde et al., 2017).

Tikuisis (1995) used a mathematical model to predict survival times under sedentary conditions in low Temp and high Wind. With maximum insulation of three loose layers, each 1 mm thick, the sedentary survival time is 18 h in combined Temp and Wind conditions close to the average recorded in our study for non-winter ascents of K2 (−20°C, Wind 14 m⋅s^–1^). The calculated survival time for typical non-winter Everest conditions (−25°C, Wind 14 m⋅s^–1^) is 12 h and for conditions similar to winter ascents of low Karakoram peaks (−40°C, Wind 14 m⋅s^–1^) survival time is only 6 h (Tikuisis, 1995). A climber forced to bivouac in winter Death Zone conditions has two to three times less survival time than in climbing season.

The level of insulation plays an important role in a climber’s ability to survive thermal stress (Tikuisis, 1995). Havenith (2010) calculated that a climber without supplemental oxygen at an altitude close to that of Everest’s summit and in non-winter conditions of Temp −25°C and Wind of 11 m⋅s^–1^, similar to those calculated in our study, needs clothing insulation of 4.5 clo to keep thermal balance. When Temp drops to −40°C, as we calculated for winter on Karakoram’s 8K peaks, and Wind is 11 m⋅s^–1^, a climber should have insulation of 6.5 clo (Havenith, 2010). Modern high-altitude clothing provides insulation of about 5.5 clo (Havenith, 2010), which does not suffice for Death Zone conditions in winter. Given our findings of extremely low Temp and high Wind in winter ascents, those planning winter expeditions should precisely calculate the insulation properties of their climbing outfits, emergency survival bags and shelters. The choice of equipment should be based on research done for emergency medicine (Oliver et al., 2016; Haverkamp et al., 2018). Nevertheless, further research is needed for survival equipment that effectively counteracts or at least ameliorates thermal stress in the Death Zone.

### Highlanders vs. Lowlanders

Our analysis of all ascents of Everest without supplemental oxygen showed that of the 64 ascents accomplished by highlanders 38% were in the 50 climbs experiencing the most severe BP. Only 18% of all 147 ascents by lowlanders were in this group. No highlanders died descending from Everest in these attempts, whereas on average every 13th successful ascent by lowlanders ended with one climber’s death on the descent in these climbs. Firth et al. (2008) analyzed all ascents of Everest, with and without oxygen support, and reported that Sherpas died more than six times less frequently than other climbers on the descent from the summit. These authors concluded that the lower incidence of death among Sherpas might be due to the better acclimatization they achieve by spending more time in higher camps, by their higher chances for rescue because Sherpas work in groups, by selection to work at high altitude, and by a genetic adaptation for altitude (Firth et al., 2008).

We observed that highlanders endured worse BP than lowlanders in non-winter ascents of Everest without oxygen. Highlanders have made the only winter ascents of Everest and K2 without oxygen: Ang Rita Sherpa of Everest, and the Magar Nirmal Purja of K2. The exceptional Ang Rita Sherpa has reached the summit of Everest nine times without oxygen (Garrido et al., 2019). Three of those were in the five ascents experiencing the lowest BP ever survived by mountaineers. Nirmal Purja was the first and only person to climb all 14 of the 8K peaks within a year (Beaumont, 2021). The fact that winter ascents of Everest and K2 without oxygen have been accomplished only by highlanders and their significantly lower incidence of deaths in the Death Zone compared with lowlanders reported in our study and in others (Firth et al., 2008) suggest that highlanders possess a superior ability to survive the extreme weather at high altitude and to tolerate higher hypoxia. Tibetans have adapted to environmental hypoxia for 500 generations (Gilbert-Kawai et al., 2014). Highlanders’ multitude of metabolic, physiological and biochemical adaptations to hypoxia are responsible for their performance at extreme altitude (Grocott and Levett, 2013; Gilbert-Kawai et al., 2014; Horscroft et al., 2017).

Reduced maximal oxygen uptake (VO_2_max) due to extremely low BP and its detrimental effect on climbing speed are the main factors limiting ascents of the highest 8K peaks without oxygen (West, 1999; Matthews et al., 2020b). The extremely high average sea-level VO_2_max of 67 ml⋅kg^−1^⋅min^−1^ of elite Sherpas reported by Garrido et al. (1997, 2019) compared with an average of 57 ml⋅kg^−1^⋅min^−1^ for lowland climbers analyzed in high-altitude scientific research (Pugh et al., 1964; West, 1983) might help explain the highlanders’ higher performance and survival at extreme altitudes. Ang Rita Sherpa had VO_2_max of 67 ml⋅kg^−1^⋅min^−1^ at the age of 53 (Garrido et al., 1997, 2019). High VO_2_max at sea level should be an important factor in selecting climbers for winter expeditions to 8K peaks.

### Limitations and Strengths

Our calculations of weather factors were not based on *in situ* measurements but on the ERA5 Reanalysis dataset, which has a spatial resolution of 0.25 degrees of latitude and longitude. When we calculated Wind we assumed there was no natural protection on the summits, such as rock formations or slopes. Yet mountain topography does affect climbers: a route on the windward side of a mountain would expose them to more extreme Wind than a route on the leeward side. Though we analyzed only successful ascents of 8K peaks, mountaineers might well have experienced more extreme conditions in expeditions that turned back because of weather breakdown before they reached the summit. Off-season attempts of 8K peaks lower than Everest and K2 might also subject climbers to more extreme weather conditions than those calculated in our study. The standard equations for WCT and FFT do not include the change in air density with altitude and therefore likely overestimate heat loss at altitude. Wind chill temperature and FFT should therefore be validated for the high-altitude low-BP environment, or a more complete human heat balance index needs to be devised.

This study provides the first complete assessment of the environmental factors that climbers have experienced in the most extreme successful ascents of 8K peaks. It develops the findings of our previous report (Szymczak et al., 2021), where we analyzed the weather extremes on the summits of Everest and K2 in the climbing and the winter season using monthly means between 1979 and 2019. This analysis extends the current knowledge of combined weather extremes in the Death Zone and should help future expeditions prepare for the environmental extremes they will encounter on 8K summits. Our study should also help climbers to interpret weather forecasts when planning a summit bid. This data will also help alpinist trainers, physiologists and researchers using environmental chambers. Knowledge of what to expect in the Death Zone in a particular season on a mountain range would help future mountaineers maximize their chances of reaching the summit while minimizing the risks of their attempts.

## Conclusion and Recommendations for Mountaineers

1. The most favorable Death Zone climatic conditions were experienced in the climbing season on the summit of K2 (July and August) and Everest (May and October), but climbing-season weather extremes in the Death Zone were more severe on Everest than on K2.
2. To find the most favorable climatic conditions, climbers planning ascents of 8K peaks without oxygen support should choose July and August for attempts of 8K peaks in Karakoram and May or October for ascents of Himalayan 8K peaks.
3. The most extreme combined environmental conditions of BP, Temp and Wind were encountered in winter and off-season ascents of Everest.
4. Climbers planning winter or off-season attempts of 8K peaks should expect worse BP, Temp and Wind in the Death Zone than in climbing season.
5. Extreme wind speed characterized winter ascents of all 8K Himalayan peaks, but severely low temperatures marked winter climbs in Karakoram.
6. Mountaineers should recognize the different characteristics of winter climatic conditions in the Death Zones of the Himalayas and Karakoram by preparing for extreme Wind in the Himalayas and severely low Temp in Karakoram.
7. Mountaineers using oxygen support survived more extreme conditions than climbers without oxygen.
8. Climbers planning winter exploration of 8K peaks should consider using oxygen support, especially when new and technically difficult routes are planned.
9. Sufficient oxygen sets should be available at the highest camps on 8K peaks for rescues during ascents and descents in the climbing and the winter seasons.
10. Expeditions to 8K peaks should regularly monitor at least BP and Temp in base and at higher camps, and also consult professional weather forecasts.

## Data Availability Statement

Publicly available datasets were analyzed in this study. This data can be found here: https://www.himalayandatabase.com/; https://www.ecmwf.int/en/forecasts/datasets/browse-reanalysis-datasets; http://www.8000ers.com/cms/k2-general-info-192.html.

## Author Contributions

RS, MN, and MS: Conceptualization. RS and MM: Methodology, Validation, and Data Curation. RS, MM, and TG: Formal Analysis. RS: Investigation, Supervision, and Writing – Original Draft Preparation. RS and MS: Resources and Visualization. RS, MN, MS, MM, and TG: Writing – Review and Editing. MS: Project Administration. RS and MN: Funding Acquisition. All authors contributed to the article and approved the submitted version.

## Conflict of Interest

The authors declare that the research was conducted in the absence of any commercial or financial relationships that could be construed as a potential conflict of interest.
